# Bioinformatic Evidence Suggesting a Dopaminergic-Related Molecular Association Between GenX Exposure and Major Depressive Disorder

**DOI:** 10.3390/toxics13121046

**Published:** 2025-12-02

**Authors:** Xiangyuan Huang, Yanyun Wang, Yuqing Zheng, Weiguang Wang, Ying Lu

**Affiliations:** 1 School of Traditional Chinese Medicine, Beijing University of Chinese Medicine, Beijing 100105, China; scy2259420@163.com (X.H.);; 2 School of Traditional Chinese Medicine, Tianjin University of Traditional Chinese Medicine, Tianjin 301617, China

**Keywords:** GenX, major depressive disorder, environmental exposure, dopaminergic dysfunction, bioinformatics

## Abstract

With the increasing global burden of major depressive disorder (MDD), identifying modifiable environmental risk factors has become a critical priority. Per- and polyfluoroalkyl substances (PFASs), characterized by environmental persistence and bioaccumulation, have been linked to elevated mental health risks. However, the potential neurotoxicity of GenX—a novel PFAS developed to replace perfluorooctanoic acid (PFOA)—and its molecular association with MDD remain unclear. In this study, peripheral blood serum transcriptomic data from the Gene Expression Omnibus (GEO) were integrated with multidimensional bioinformatics analyses to elucidate molecular mechanisms connecting GenX exposure with MDD. Four hub genes (UCP2, AKR1B1, TP53, and F5) were identified, showing strong combined diagnostic performance (AUC = 0.925). Functional enrichment and immune infiltration analyses revealed their involvement in energy metabolism, oxidative stress, and immune-coagulation regulation. Molecular docking and dynamics simulations further confirmed stable interactions between GenX and these proteins, providing structural support for their mechanistic roles. Although classical dopaminergic markers (TH, SLC6A3, DRD1–5) were not detected in the serum-derived transcriptomes, the identified hub genes may still affect dopaminergic function indirectly by modulating metabolic, oxidative stress, and inflammatory/coagulation pathways, thereby influencing MDD susceptibility. This study provides the first integrated transcriptomic and structural evidence linking GenX to psychiatric risk, proposing a novel “GenX-dopamine-MDD” framework for understanding pollutant-mediated neuropsychiatric mechanisms.

## 1. Introduction

Major depressive disorder (MDD) is a common psychiatric condition characterized by persistent depressive episodes lasting at least two weeks. These episodes are often accompanied by disturbances in mood, interest, cognition, and autonomic function [1]. According to the Global Burden of Disease (GBD) study, the number of MDD cases increased by nearly 60% between 1990 and 2019, along with rises in morbidity and disability-adjusted life years (DALYs), highlighting its growing global impact [2]. MDD not only impairs social functioning and quality of life but is also associated with a high risk of suicidal ideation and behavior, representing a significant public health concern [3]. Given this substantial burden, identifying modifiable risk factors for MDD is a critical priority. Among these, environmental factors have emerged as important contributors. Increasing evidence suggests that exposure to environmental pollutants may affect mental health. While links between certain air pollutants or heavy metals and MDD have been reported, the neuropsychiatric effects of many newly developed industrial chemicals remain poorly understood [4].

Per- and polyfluoroalkyl substances (PFASs) are a class of persistent organic pollutants, including perfluorooctanoic acid (PFOA) and perfluorooctanesulfonate (PFOS). These compounds are widespread in the environment and are highly bioaccumulative [5]. Their potential neurotoxic effects have drawn increasing attention. Several epidemiological studies have suggested possible links between PFAS exposure and mental health outcomes. For example, analysis of NHANES data indicated a positive association between serum PFAS levels and depressive symptoms in adults, while maternal exposure during pregnancy was also associated with an increased risk of depression [6,7,8]. Most of these studies, however, have focused on traditional PFASs, and the neuropsychiatric effects of newer alternatives remain largely unknown. GenX, formally known as hexafluoropropylene oxide dimer acid (HFPO-DA), is a recently developed PFAS initially introduced as a substitute for PFOA and widely used in industrial applications. Due to its shorter carbon chain, GenX was originally considered a “safer” alternative with lower bioaccumulation potential [9]. In various regions of China, the estimated daily intake (EDI) of GenX through diet ranges from 2.33 to 3.96 ng/kg body weight per day, corresponding to 0.78–1.32 times the oral reference dose (RfD = 3.0 ng/kg bw/day) established by the U.S. Environmental Protection Agency. Notably, in certain areas, including North, Central, and South China, the dietary intake of GenX exceeds the RfD, indicating potential health risks associated with exposure [10]. These findings suggest that GenX is an emerging environmental contaminant associated with increasing human exposure. Multiple studies have reported that GenX exhibits hepatotoxicity, neurotoxicity, and adverse effects on the reproductive system [11,12,13]. Of particular concern for the nervous system, GenX exposure has been shown to induce mitochondrial dysfunction and neuroinflammation in astrocytes, along with downregulation of dopaminergic (DA) neuronal markers [14]. Oxidative stress appears to be a key pathological mechanism underlying DA neuronal degeneration. DA neurons are particularly susceptible to oxidative stress due to their intrinsic tendency toward auto-oxidation and subsequent generation of reactive oxygen species (ROS). Under conditions of oxidative stress and neuroinflammation, these neurons are more vulnerable to functional impairment or degeneration [15,16]. Liu et al. reported that GenX exposure increased ROS production and induced oxidative stress, confirming its inhibitory effects on DA neurons [17]. These findings indicate that GenX may affect pathways involved in neuronal energy metabolism and oxidative stress, both of which play critical roles in emotional regulation. Reduced DA activity within the mesencephalic system is considered a key neurobiological feature of MDD [18]. In the well-established unpredictable chronic mild stress (CMS) rat model of depression, DA neuronal ensemble activity is significantly diminished, a process associated with inhibition of the basolateral amygdala (BLA)–ventral pallidum (VP) pathway [19]. Based on these observations, we hypothesize that GenX exposure may contribute to MDD by affecting dopaminergic neurons and their downstream signaling networks.

Although previous studies have suggested that GenX can affect dopamine homeostasis, its precise molecular mechanisms in humans remain largely unclear. Direct bioinformatic evidence supporting a potential molecular association network between GenX and MDD is currently lacking. Traditional epidemiological and animal studies are limited in their ability to elucidate complex molecular relationships between environmental exposures and psychiatric disorders. Consequently, integrated bioinformatics analyses provide a useful approach. By combining transcriptomic datasets with GenX target gene profiles, applying machine learning to identify hub genes, and examining immune infiltration patterns to assess changes in the tissue microenvironment, it is possible to explore potential shared molecular networks and signaling pathways between GenX and MDD. To support the plausibility of these computational predictions, we further evaluated the binding modes and stability of GenX with key hub proteins using molecular docking and molecular dynamics (MD) simulations. This approach offers structural-level evidence supporting bioinformatic predictions and indicates that GenX could potentially affect dopaminergic-related pathways by interacting with the structure or function of these proteins. In this study, we combined transcriptomic analysis with molecular modeling to explore possible dopaminergic-related molecular links between GenX and MDD and to identify core hub genes that may be involved. These results provide preliminary insights that could guide future research into the mechanisms by which environmental pollutants might contribute to psychiatric disorders.

## 2. Materials and Methods

### 2.1. Acquisition of MDD, Dopamine, and GenX-Related Genes

Gene expression datasets related to MDD (GSE52790 and GSE76826) were retrieved from GEO database (National Center for Biotechnology Information, Bethesda, MD, USA). The datasets were integrated following batch correction procedures to ensure comparability across studies. All expression matrix files were processed and gene identifiers standardized, after which the overlapping genes among datasets were extracted to maintain data consistency. The preprocessed matrices were merged, and duplicate gene entries were averaged using the avereps function from the limma package (Bioconductor; Roswell Park Comprehensive Cancer Center, Buffalo, NY, USA), with batch information recorded. Batch effects were adjusted using the ComBat function from the sva package (Bioconductor; Buffalo, NY, USA) based on an empirical Bayesian framework, preserving biological variability. The efficiency of batch correction was visually assessed using principal component analysis (PCA), with pre- and post-correction distributions compared via ggplot2 scatter plots. Differentially expressed genes (DEGs) were subsequently identified using the limma package, applying thresholds of adjusted *p* < 0.05 and |log_2_ fold change| > 0.25. Differential expression patterns were visualized using the pheatmap package (Raivo Kolde, Estonia). For GenX-related genes, chemical–gene interactions associated with GenX were obtained from the Comparative Toxicogenomics Database (CTDbase; North Carolina State University, Raleigh, NC, USA; https://ctdbase.org/, accessed on 14 September 2025). Dopamine-related genes were retrieved from GeneCards (The Weizmann Institute of Science, Rehovot, Israel; https://www.genecards.org, accessed on 14 September 2025) using the search terms “dopamine”, “dopamine receptor”, and “dopaminergic”, retaining genes with relevance scores above the median. Finally, an intersection analysis among the GenX-related, dopamine-related, and MDD-related gene sets was performed to identify shared candidate genes.

### 2.2. Hub Gene Screening Based on Machine Learning

To identify hub genes with core biological relevance, a multidimensional screening framework integrating two complementary machine learning algorithms—Least Absolute Shrinkage and Selection Operator (LASSO) regression and Support Vector Machine–Recursive Feature Elimination (SVM-RFE; TU Wien, Vienna, Austria)—was established based on the intersecting gene set. The combined use of these algorithms improved the robustness of feature selection: LASSO emphasized model sparsity and minimized overfitting, whereas SVM-RFE enhanced the discrimination of boundary genes.

LASSO regression was implemented to determine the optimal regularization parameter (λ) through five-fold cross-validation. In parallel, SVM-RFE assessed model performance across varying feature numbers using five-fold cross-validation, with the final feature count selected according to the one-standard-error rule. The intersection of genes identified by both methods was defined as the final hub gene set (Table 1).

### 2.3. Diagnostic Performance Evaluation of Hub Genes

To evaluate the diagnostic efficiency and functional significance of the identified hub genes, differential expression and correlation analyses were conducted. Box plots were generated to visualize the expression profiles of hub genes between MDD and control groups, and significance was determined using the Wilcoxon rank-sum test. Pearson correlation analysis was used to construct gene co-expression networks, and correlation strengths were visualized through heatmaps. Diagnostic performance was further assessed in two ways: ① Receiver operating characteristic (ROC; Université de Lyon, Lyon, France) curves were plotted for individual genes, and the area under the curve (AUC) values were calculated to quantify single-gene diagnostic power; ② A multivariate logistic regression model integrating all hub genes was constructed, and the combined diagnostic efficacy was evaluated by analyzing regression coefficients and calculating the composite AUC value.

### 2.4. Genome-Wide Visualization and Functional Enrichment of Hub Genes

To visualize the genomic distribution patterns of hub genes and determine whether their dysregulation reflects localized chromosomal events or systemic transcriptional regulation, we performed genome-wide mapping based on the human reference genome (hg38). A circular genome plot was generated to display the chromosomal locations of the target genes, while a Manhattan plot illustrated their genome-wide statistical distribution. To further elucidate the biological roles and signaling pathways associated with these hub genes, Gene Ontology (GO) enrichment and Kyoto Encyclopedia of Genes and Genomes (KEGG) pathway analyses were conducted, with a significance threshold set at *p* < 0.05.

### 2.5. Immune Infiltration Correlation Analysis of Hub Genes

The CIBERSORT (Stanford University, Stanford, CA, USA) algorithm was applied to assess immune cell infiltration using batch-corrected transcriptomic data. Based on the LM22 immune cell signature matrix, the relative proportions of 22 immune cell subsets were estimated via linear support vector regression. The number of permutation tests was set to 1000, and samples with *p* < 0.05 were retained for downstream analyses. Stacked bar charts were used to depict the immune cell composition across groups, while box plots compared the relative abundance of each immune cell subtype between control and disease groups. The Wilcoxon rank-sum test was applied to evaluate statistical significance. A correlation heatmap of immune cell populations was generated using Spearman’s correlation coefficients to assess intercellular relationships. Associations between hub genes and immune infiltration were analyzed using the linkET package (developed by Yige Sun, Shanghai, China), calculating Spearman’s correlation coefficients between gene expression and immune cell abundance. Finally, chord diagrams were constructed to visualize gene–immune cell interaction networks, with correlation strength and significance represented by absolute correlation coefficient values and *p*-values.

### 2.6. GeneMANIA-Based Functional Association and Biological Clustering of Hub Genes

To systematically expand the functional gene repertoire associated with the hub genes, we employed GeneMANIA-based network analysis. For each hub gene, GeneMANIA identifies functionally related genes through three major interaction types: co-expression, genetic interaction, and physical protein–protein interaction. This integrative approach enhances the detection of biologically relevant gene networks by capturing complementary functional associations reflecting shared pathways and protein complexes. The subnetworks constructed around each hub gene were merged to generate an expanded functional gene network, termed the GeneMANIA-based Functionally Associated Expanded Hub Gene dataset (GM-Hub). This enriched network served as the foundation for biological functional annotation. GO and KEGG enrichment analyses were subsequently performed on the GM-Hub genes to identify significantly enriched biological processes and signaling pathways. To reduce network noise and extract interpretable core functional modules, we performed functional clustering using the MCODE plugin in Cytoscape (version 2.10.3; Cytoscape Consortium, San Diego, CA, USA). The analysis was conducted with the following parameters: K-Core = 2, node fraction cut-off = 0.2, degree cut-off = 2, clustering method = “haircut,” and maximum depth = 100 (default). Each resulting cluster was treated as an independent subnetwork and subjected to GO and KEGG enrichment analyses to elucidate its biological and functional relevance.

### 2.7. Molecular Docking and Molecular Dynamics Simulation

To verify the binding affinity and interaction stability between GenX and hub gene-encoded proteins, molecular docking and molecular dynamics (MD) simulations were performed. The three-dimensional structure of GenX was retrieved from the PubChem database (https://pubchem.ncbi.nlm.nih.gov/, accessed on 14 September 2025) in sdf format, while the crystal structures of target proteins were obtained from the Protein Data Bank (PDB) (https://www.rcsb.org/, accessed on 14 September 2025) in pdb format. Both proteins and ligands were dehydrogenated, hydrogenated, and structurally optimized using PyMOL 2.6.0, and binding pocket parameters were automatically identified using the Getbox plugin. Protein and ligand structures were converted to pdbqt format in AutoDock Tools 1.5.6, and molecular docking was carried out using AutoDock Vina 1.1.2(the Scripps Research Institute, La Jolla, CA, USA). Binding affinity (kcal/mol) was used to assess ligand–protein binding strength. Docking poses were visualized in PyMOL to analyze hydrogen bonding, hydrophobic interactions, and conformational changes within the binding pocket. To further evaluate binding stability, 100 ns molecular dynamics simulations were performed on four protein–ligand complexes using GROMACS 2024.4(KTH Royal Institute of Technology, Stockholm, Sweden). The system was parameterized with the Amber99SB-ILDN force field for proteins, while ligand topologies were generated using the Sobtop tool based on the GAFF2 force field. Each complex was solvated in a cubic water box (SPC/E model) with periodic boundary conditions, extending 1.2 nm beyond the solute in all directions. Appropriate Na^+^ and Cl^−^ ions were added to neutralize the system. Energy minimization was performed using the steepest descent algorithm, followed by equilibration under NVT and NPT ensembles for 100 ps each. The 100 ns production run was carried out at 300 K and 1 bar with a 2 fs time step. Long-range electrostatic interactions were calculated using the Particle Mesh Ewald (PME) method, while van der Waals interactions were treated using a force-switch function with a cutoff radius of 1.0 nm. Trajectory data were saved every 50,000 steps for subsequent analysis. System stability was assessed by evaluating the root-mean-square deviation (RMSD), radius of gyration (Rg), solvent-accessible surface area (SASA), and root-mean-square fluctuation (RMSF) of each complex. The Free Energy Landscape (FEL) was further constructed to identify the energy-minimum conformational states of the complexes, providing insight into their dynamic stability and conformational preferences.

## 3. Results

### 3.1. Intersection of MDD, Dopamine and GenX-Related Genes

**Figure 1:**
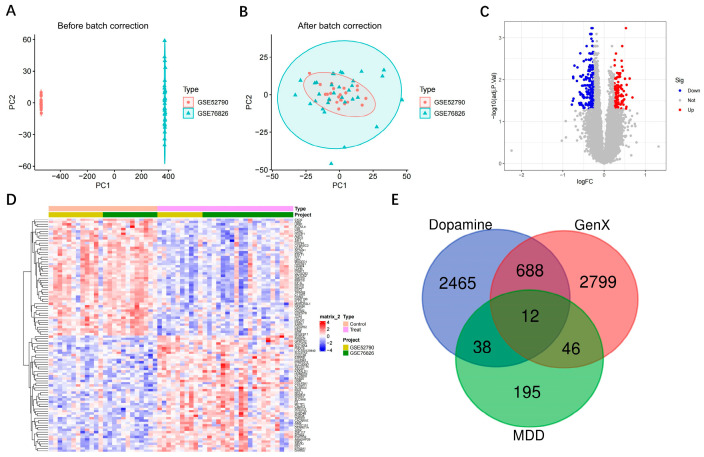
Acquisition and integrated analysis results for MDD, dopamine, and GenX-related genes. (**A**) Principal component analysis before batch correction; (**B**) Principal component analysis after batch correction; (**C**) Volcano plot of differentially expressed genes; (**D**) Heatmap of differentially expressed genes; (**E**) Venn diagram of intersecting genes.

Integration and batch correction of the datasets demonstrated effective removal of batch effects. Prior to correction (Figure 1A), the two datasets GSE52790 and GSE76826 showed distinct separation along the PC1 axis, with GSE52790 samples clustering to the left and GSE76826 samples to the right, indicating a pronounced batch effect. Following batch correction (Figure 1B), the ComBat algorithm successfully eliminated the batch effects, resulting in well-integrated and overlapping sample distributions within the principal component space. The volcano plot (Figure 1C) illustrates the distribution of upregulated (red) and downregulated (blue) genes, with the majority of differentially expressed genes exhibiting strong statistical significance. The heatmap (Figure 1D) visualizes the expression profiles of these genes across all samples, showing clear separation between control and disease groups, thereby confirming the reliability of the differential expression analysis. Intersection analysis among the multi-source gene sets (Figure 1E) revealed that the dopamine-related gene set contained 3165 genes, the GenX-related gene set included 3533 genes, and the MDD differential gene set comprised 291 genes. The intersection of these three datasets identified 12 shared genes, representing the potential molecular links between GenX exposure, dopaminergic regulation, and MDD (Figure 1) (Table 2).

### 3.2. Identification of Hub Gene Screening Based on Machine Learning

**Figure 2:**
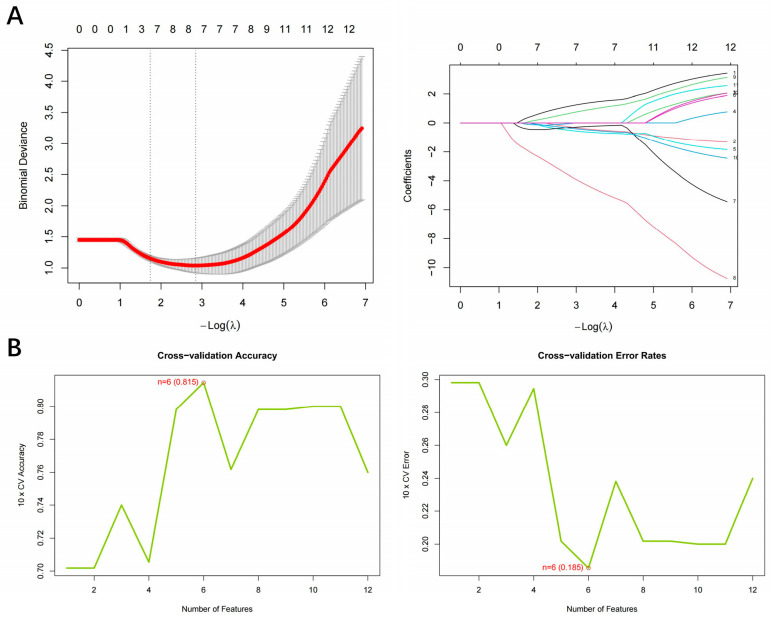
Machine learning-based hub gene screening results. (**A**) LASSO regression analysis results. The left panel shows the cross-validation curve for selecting the optimal λ value, where the red curve represents the model deviance across λ values and the gray shaded region indicates the standard error. The right panel displays the LASSO coefficient profiles for all input genes; each colored line represents the coefficient trajectory of an individual gene across λ values. (**B**) SVM-RFE analysis results. The left panel shows cross-validation accuracy across different numbers of selected features, while the right panel shows the corresponding cross-validation error rate. The green lines represent accuracy and error trends, and red labels mark the optimal feature number identified by the model.

By integrating LASSO regression and SVM-RFE machine learning algorithms, we successfully identified hub genes with core biological significance. During the five-fold cross-validation in LASSO regression analysis, the binomial deviance reached its minimum when −log(λ) ≈ 3, at which point the model retained 10 feature genes with non-zero coefficients. The regression coefficient trajectory demonstrated that as the regularization strength increased, most coefficients gradually approached zero, whereas key genes maintained stable non-zero coefficients. It highlighted the sparsity advantage of the LASSO algorithm in feature selection (Figure 2A). SVM-RFE analysis revealed a non-linear trend in model performance as the number of features varied. Cross-validation accuracy peaked when the number of features reached five, remaining relatively stable between seven and ten features. Based on the one-standard error rule, seven features were ultimately selected as the optimal subset in the SVM-RFE model (Figure 2B). The intersection of the two algorithmic outputs identified four hub genes—UCP2, AKR1B1, TP53, and F5. These genes simultaneously satisfied the sparsity criteria recognized by LASSO regression and the boundary optimization conditions of SVM-RFE, ensuring both the robustness and biological reliability of the screening results (Figure 2).

### 3.3. Diagnostic Performance and Associated Features of Hub Genes

**Figure 3:**
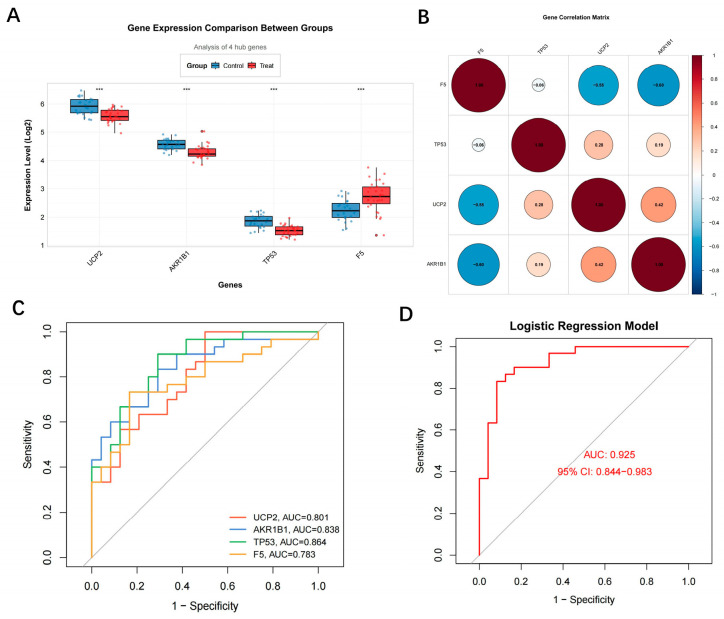
Evaluation of diagnostic efficacy for hub genes. (**A**) Differential expression analysis of hub genes; (**B**) Heatmap of hub gene correlation analysis; (**C**) Single-gene ROC curve analysis; (**D**) ROC curve for the multi-gene combined diagnostic model.

Comprehensive evaluation of the diagnostic efficacy of the four identified hub genes revealed their strong potential for disease discrimination. Except for F5, which was upregulated, the remaining three genes were significantly downregulated in MDD samples (Figure 3A). Correlation analysis revealed a complex co-expression network among the hub genes. UCP2 exhibited negative correlations with all three other genes, with the strongest negative association observed with TP53. In contrast, AKR1B1 and F5, as well as TP53 and F5, showed positive correlations, suggesting potential participation in shared biological pathways (Figure 3B). At the single-gene level, ROC curve analysis demonstrated that all four hub genes possessed substantial diagnostic value, with TP53 showing the highest AUC among them (Figure 3C). When combined into a multivariate logistic regression model, the hub gene panel achieved an AUC of 0.925, surpassing the diagnostic performance of individual genes. This indicates excellent clinical diagnostic potential and further validates the integrated machine-learning screening strategy (Figure 3D) (Figure 3).

### 3.4. Genome-Wide Visualization and Functional Enrichment of Hub Genes

**Figure 4:**
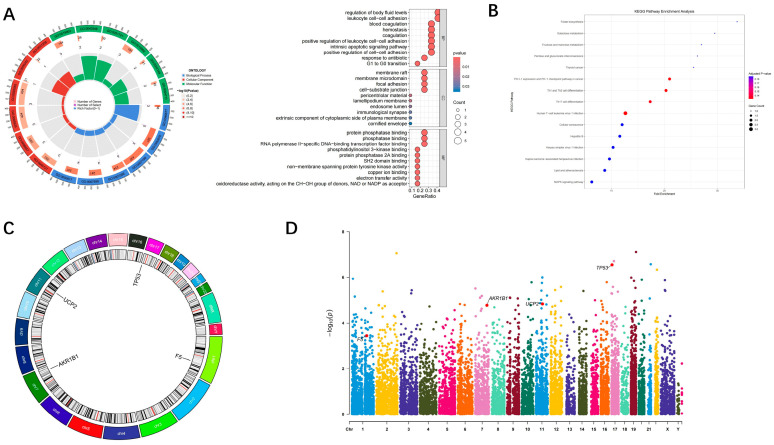
Genome-wide visualization and functional enrichment analysis results for the hub genes. (**A**) Circular genome plots; (**B**) KEGG pathway enrichment analysis results; (**C**) GO functional enrichment analysis pie chart; (**D**) Genome-wide association Manhattan plot.

GO functional enrichment analysis revealed that the hub genes were primarily involved in immune regulation and coagulation-related biological processes (Figure 4A). For biological processes (BP), significantly enriched terms included humoral regulation, leukocyte-cell adhesion, and blood coagulation regulation (*p* < 0.05). For molecular functions (MF), enrichment was observed in protein binding and kinase activity modulation, while cellular component (CC) analysis indicated predominant localization of hub gene products to the cell membrane and cytoplasm. KEGG pathway enrichment further identified key signaling networks such as the complement and coagulation cascade, apoptosis, and p53 signaling pathways (Figure 4B). These results suggest that the hub genes may contribute to MDD onset and progression by modulating cell death, immune responses, and coagulation homeostasis. Genome-wide visualization revealed distinct chromosomal distributions of the hub genes. Circular genome mapping showed that UCP2 is located on chromosome 11, AKR1B1 on chromosome 7, TP53 on chromosome 17, and F5 on chromosome 1. Their distribution across different chromosomes implies that their dysregulation likely stems from systemic transcriptional regulatory mechanisms rather than local chromosomal aberrations (Figure 4C). The Manhattan plot further confirmed the genome-wide statistical significance of these genes, with TP53 and AKR1B1 exhibiting the highest significance peaks within their respective chromosomal regions (Figure 4D) (Figure 4).

### 3.5. Analysis of Hub Gene Correlation with Immune Infiltration

**Figure 5:**
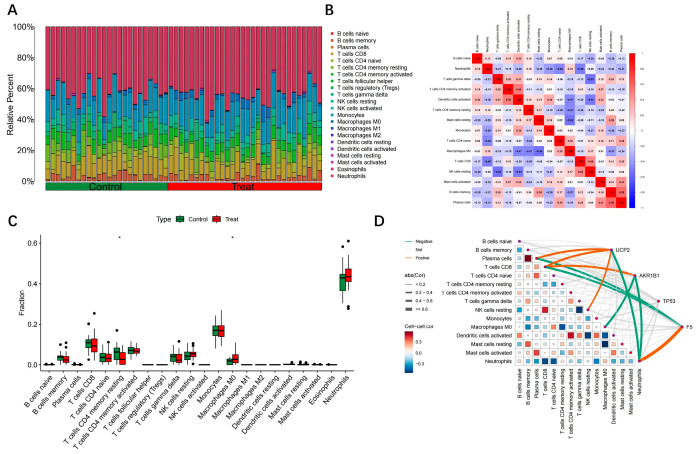
Analysis results of Hub gene-immune cell infiltration correlation. (**A**) Stacked bar chart of immune cell infiltration accumulation; (**B**) Heatmap of immune cell correlation; (**C**) Box plot of intergroup differences in immune cells. * indicates a statistically significant difference (p < 0.05).; (**D**) Chord diagram of hub gene-immune cell association.

Immune cell infiltration analysis revealed substantial differences in immune microenvironmental composition between the disease and control groups. Using the CIBERSORT algorithm, the relative abundances of 22 immune cell subpopulations were successfully deconvoluted, with all samples yielding permutation test *p*-values < 0.05, confirming the statistical reliability of the deconvolution results. The stacked bar chart illustrates clear differences in immune cell composition between the two cohorts. The disease group exhibited markedly elevated proportions of M0 macrophages, resting NK cells, and neutrophils, while the control group showed higher proportions of memory B cells, CD8^+^ T cells, and regulatory T cells (Tregs) (Figure 5A). Comparative analysis of immune cell abundance between groups (Figure 5B) identified several immune subpopulations with significant differential infiltration. Notably, M0 macrophages were markedly increased in the disease group (median ~0.45 vs. 0.20), accompanied by a significant rise in resting NK cells. In contrast, memory B cells, CD8^+^ T cells, and follicular helper T cells were significantly reduced, suggesting that adaptive immune function may be suppressed under disease conditions. Correlation network analysis of immune cell subsets revealed complex intercellular regulatory relationships. The correlation heatmap demonstrated strong positive correlations primarily among functionally related cell subsets, such as various memory T-cell populations. Conversely, M0 and M1 macrophages exhibited predominantly negative correlations with lymphocyte populations, reflecting a dynamic equilibrium within the immune microenvironment (Figure 5C). Hub gene–immune infiltration correlation analysis, visualized via chord diagrams (Figure 5D), revealed distinct association patterns between the four hub genes and specific immune cell subsets. UCP2 showed strong positive correlations with M0 macrophages and neutrophils, but negative correlations with memory B cells and CD8^+^ T cells. AKR1B1 was positively associated with regulatory T cells and memory CD4^+^ T cells. TP53 displayed complex associations with multiple lymphocyte subsets, most notably a strong positive correlation with follicular helper T cells. F5 primarily correlated with coagulation-associated platelet function and monocyte infiltration. Collectively, these findings suggest that hub genes may contribute to disease pathogenesis through modulation of the immune microenvironment (Figure 5).

### 3.6. GeneMANIA Functional Association and Biological Function Clustering of Hub Genes

**Figure 6:**
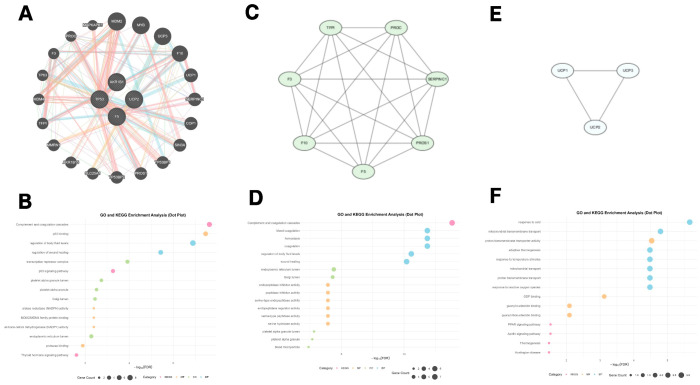
GeneMANIA functional association and biological function clustering analysis results for hub genes. (**A**) GeneMANIA functional association network diagram; (**B**) GO and KEGG enrichment analysis results for the GM-Hub gene set; (**C**) MCODE clustering sub-network 1; (**D**) Functional enrichment analysis of clustering sub-network 1; (**E**) MCODE clustering sub-network 2; (**F**) Functional enrichment analysis of clustering sub-network 2.

Construction of the GeneMANIA functional association network expanded the set of genes functionally related to the identified hub genes. Based on three major interaction types—co-expression, genetic interaction, and protein–protein interaction—an extended gene network exhibiting strong functional associations was established. As shown in Figure 6A, TP53 emerged as a central hub, forming dense connections with key regulatory genes such as MDM2, MYC, and UCP1, underscoring its pivotal role as the “guardian of the genome.” AKR1B1 primarily formed metabolic modules with genes including UGCG and SERPINC1, reflecting its roles in glucose metabolism and oxidative stress regulation. F5 clustered with genes such as F10, F3, and THBD within a coagulation-related regulatory network, highlighting its essential function in hemostasis. UCP2 formed distinct functional clusters with genes associated with mitochondrial activity and energy metabolism. The colors and line thicknesses within the network represent varying interaction types and strengths. Using MCODE clustering analysis, several key functional modules of biological significance were identified (K-Core = 2, node score cutoff = 0.2). Three principal sub-networks were extracted. The first sub-network (Figure 6C), centered around TP53, formed a tightly connected module consisting of seven genes, including PROC, F5, and SERPINC1, primarily involved in cell cycle regulation and DNA damage response. The second sub-network (Figure 6E), centered on the mitochondrial uncoupling protein family (UCP1, UCP2, UCP3), formed a compact triangular structure, reflecting their synergistic function in regulating mitochondrial dynamics and bioenergetics.

Functional enrichment analysis of the expanded gene set (Figure 6B) revealed significant enrichment across several key biological processes. GO analysis indicated that the genes were predominantly involved in complement and coagulation cascades, p53 binding, and humoral regulation, with p53 binding showing the highest enrichment significance (approximately –log_10_ FDR = 6, gene count = 5). Molecular function terms were enriched for protein binding, enzyme activity regulation, and NADPH oxidase activity. KEGG pathway analysis further confirmed the enrichment of metabolic and signaling pathways, including thyroid hormone signaling, peroxisome biogenesis, and thermogenesis. Functional enrichment of the TP53-centered cluster (Figure 6D) highlighted coagulation-associated processes such as blood coagulation, hemostasis, fibrinolysis regulation, and platelet aggregation—reflecting its critical role in vascular homeostasis. Meanwhile, enrichment analysis of the UCP family cluster (Figure 6F) emphasized bioenergetic processes, including mitochondrial inner membrane localization, protein transmembrane transport, and respiratory chain activity, underscoring its role in cellular energy regulation. These distinct yet interconnected functional modules provide valuable insight into the cooperative mechanisms by which hub genes modulate disease-related biological processes (Figure 6).

### 3.7. Molecular Docking and Molecular Dynamics Simulations

**Figure 7:**
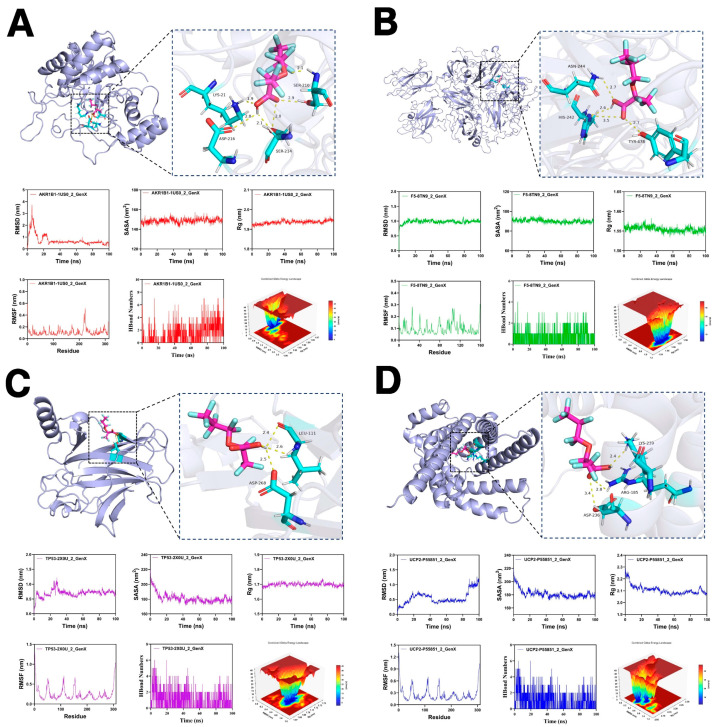
Molecular docking and molecular dynamics simulation results of GenX with hub genes. (**A**) Binding mode of GenX with AKR1B1 and the corresponding 100 ns molecular dynamics simulation results. (**B**) Binding mode of GenX with F5 and the corresponding 100 ns molecular dynamics simulation results. (**C**) Binding mode of GenX with TP53 and the corresponding 100 ns molecular dynamics simulation results. (**D**) Binding mode of GenX with UCP2 and the corresponding 100 ns molecular dynamics simulation results.

Molecular docking results demonstrated that GenX can form stable binding conformations with all four core proteins. Among these, GenX exhibited the lowest binding energy with AKR1B1 (−8.2 kcal/mol), indicating the strongest binding affinity. Visualization showed that GenX interacts with key residues of AKR1B1 such as SER210, TYR49, and ASN161, through hydrogen bonding, complemented by hydrophobic interactions that further stabilize the complex. The binding energies for F5, TP53, and UCP2 were −7.3, −5.8, and −6.4 kcal/mol, respectively, indicating moderate-to-strong affinities. GenX formed hydrogen bonds with ASN268, HIS240, and THR278 at the F5 binding site; with ASP208 and LEU111 in TP53; and with LYS276 and ASP237 in UCP2, forming a stable hydrogen-bonding network in each complex. During 100 ns molecular dynamics simulations, the RMSD values of all four complexes fluctuated within 2.0 nm, indicating equilibrium and overall structural stability. The AKR1B1-GenX complex exhibited the most stable trajectory (RMSD ≈ 1.2 nm), with consistent Rg and SASA values, suggesting minimal conformational changes or solvent exposure after binding. The F5-GenX and TP53-GenX complexes showed mild fluctuations in the late simulation phase but remained generally stable. The UCP2-GenX complex exhibited a single deep energy basin on the free energy landscape (FEL) and maintained an RMSD around 1.0 nm, reflecting a highly stable conformation. RMSF analysis indicated minimal fluctuation in the active pocket region of AKR1B1 (residues 120–160), suggesting GenX binding stabilizes this region. Conversely, moderate flexibility was observed near the TP53 binding site, possibly reflecting its stress-responsive structural dynamics. Overall, these results demonstrate that GenX possesses strong multi-target binding potential with favorable thermodynamic and conformational stability across all four hub proteins, suggesting that GenX may modulate MDD-related molecular pathways through simultaneous interaction with multiple targets (Figure 7) (Table 3).

## 4. Discussion

The process of industrialization, while generating substantial economic benefits, has also led to the continuous emergence of novel environmental pollutants, raising growing global concern about their potential impact on public health [20]. GenX, a replacement for traditional PFOA, has become widely used and detected across multiple environmental matrices. However, its potential neurotoxicity, particularly its possible association with major mental disorders such as MDD, remains poorly understood. In this study, we identified four key hub genes (UCP2, AKR1B1, TP53, and F5) using an integrated framework that combined multi-dataset transcriptomics, machine learning algorithms, immune infiltration profiling, and functional network clustering. The results suggest that GenX exposure may be associated with molecular changes relevant to MDD. Our findings indicate that GenX might potentially affect multiple biological processes, including energy metabolism imbalance, increased oxidative stress, and immune-coagulation alterations, which could indirectly influence dopaminergic neuronal function and potentially contribute to MDD susceptibility. Importantly, this study does not consider dopaminergic dysfunction a definitive pathway; rather, it is presented as a plausible hypothesis that may be indirectly influenced by inflammation, metabolic disturbances, and coagulation abnormalities.

Multidimensional algorithmic screening and cross-validation identified four genes with distinct expression changes in MDD: UCP2, AKR1B1, and TP53 were significantly downregulated, whereas F5 was upregulated. These genes exhibited strong diagnostic performance, with a combined AUC of 0.925, highlighting their potential clinical relevance. Functionally, they have distinct yet interconnected roles that converge on mitochondrial metabolism, oxidative stress regulation, and neuroinflammatory balance. UCP2, a mitochondrial uncoupling protein located in the inner mitochondrial membrane, primarily regulates proton leakage, modulates ATP synthesis efficiency, and scavenges reactive oxygen species (ROS). Its downregulation may indicate reduced energy supply to dopaminergic neurons and weakened oxidative stress defense [21]. Leena et al. reported that UCP2 influences CD8^+^ T-cell expansion by reducing glycolysis and fatty acid synthesis through inhibition of mitochondrial ROS generation [22]. In a chronic mild stress (CMS) model of depression characterized by anhedonia, UCP2-deficient mice showed exacerbated depressive behaviors, whereas UCP2 overexpression produced antidepressant effects by suppressing the ROS-thioredoxin-interacting protein (TXNIP)–NLRP3 pathway in astrocytes [23]. These findings suggest that UCP2 plays a critical role in maintaining dopaminergic neuronal energy homeostasis and redox balance, with its downregulation representing a potential molecular node through which GenX may affect dopaminergic signaling. AKR1B1, a member of the aldehyde-ketone reductase family, participates in glucose metabolism, particularly via the polyol pathway and redox regulation. Its downregulation may lead to impaired glucose metabolism and reduced endogenous antioxidant defenses, thereby promoting inflammatory responses [24]. Evidence also links AKR1B1 to diabetic complications, elevated inflammatory cytokine levels, and mood disorders [25,26,27]. Given that metabolic dysfunction and immune activation frequently coexist in MDD [27,28], AKR1B1 downregulation may represent a molecular link between metabolic and inflammatory dysregulation. Our results suggest that GenX exposure could reduce AKR1B1 expression, potentially promoting inflammation, impairing dopamine synthesis, and increasing susceptibility to depression. TP53, one of the most frequently mutated genes in human cancers, regulates DNA damage repair, cell cycle progression, and apoptosis. Its downregulation in the central nervous system may compromise neuronal DNA repair and cell cycle control, potentially affecting the stability of dopaminergic neurons [29,30]. Evidence indicates that the minor allele 72C of TP53 confers protective effects against MDD, whereas TP53 downregulation may promote inflammation-associated MDD onset [31,32]. F5, a key component of the prothrombinase complex, was significantly upregulated in this study, indicating activation of the coagulation system. Increasing evidence shows that patients with depression often exhibit coagulation abnormalities and peripheral inflammation, which can disrupt the blood–brain barrier and amplify central nervous system inflammation, contributing to MDD pathogenesis [33,34]. The upregulation of F5 aligns with enrichment results for complement and coagulation cascade pathways. Together, these four hub genes suggest a potential synergistic pathological network involving apoptosis, energy metabolism, inflammation, and coagulation.

GO and KEGG enrichment analyses further supported the functional convergence of the hub genes in complement and coagulation cascades, p53 signaling, and humoral regulation, with particular emphasis on the coagulation system. Dysregulation of the immune-coagulation axis has been implicated in MDD progression through disruption of the blood–brain barrier and activation of microglia [34,35]. Dopaminergic damage and coagulation abnormalities may co-occur within a shared pathological context characterized by inflammation and oxidative stress. A review on Parkinson’s disease (PD), a disorder primarily marked by dopaminergic neuron degeneration, reported that patients often exhibit a hypercoagulable state, including elevated fibrinogen, impaired fibrinolysis, and platelet dysfunction [36]. The co-clustering of TP53 and F5 within the same functional subnetwork suggests potential cooperative involvement in coagulation and immune regulation. UCP2, meanwhile, clustered with family members such as UCP1 and UCP3, indicating a role in disease development through modulation of energy metabolism. The enrichment of the p53 pathway aligns with TP53 downregulation, suggesting that impaired DNA repair and stress responses may represent additional mechanisms through which GenX could exert neurotoxic effects. Immune infiltration analysis provided further mechanistic insights. Hub genes were positively correlated with M0 macrophages and neutrophils but negatively correlated with adaptive immune populations such as memory B cells and CD8^+^ T cells. This pattern reflects an enhanced pro-inflammatory state and weakened adaptive immunity characteristic of MDD samples. This observation is consistent with previous reports that overactivation of the monocyte-macrophage system leads to secretion of pro-inflammatory factors, including IL-1β, IL-6, and TNF-α. These factors can cross or compromise the blood–brain barrier and activate central microglia, thereby amplifying neuroinflammation. Concurrently, reductions in adaptive immune cells may weaken immune surveillance and regulatory functions, further exacerbating inflammatory imbalance [37]. Immune and inflammatory abnormalities can also influence dopaminergic signaling. The pro-inflammatory factor MIF, secreted by microglia, may amplify neuroinflammatory responses through the TLR4/MyD88/TRAF6 pathway, whereas downregulation of MIF has been shown to reduce neuroinflammation and protect midbrain dopaminergic neurons [38]. Additionally, studies using maternal immune activation (MIA) models suggest that inflammation can affect the development of the endocannabinoid system and synaptic plasticity, thereby altering the firing patterns of VTA dopaminergic neurons [39]. These observations indicate that GenX exposure could potentially modulate these hub genes, promote macrophage-mediated inflammatory responses, disrupt immune homeostasis, and trigger a cascade of neuroinflammatory events, indirectly impacting dopaminergic function. Clinical evidence shows that patients with MDD often exhibit elevated peripheral pro-inflammatory factors. For instance, TNF-α and IL-6 levels are significantly higher in MDD patients than in healthy controls, while high-mobility group protein 1 (HMGB1) expression is markedly reduced. These inflammatory markers are associated with disease severity and may have potential diagnostic value [40]. Another study highlighted the heterogeneity of inflammatory profiles within MDD phenotypes, showing that patients with anhedonia exhibited stronger immune activation, as indicated by elevated IL-6, IL-10, TNF-α, and complement factor H (CFH), which correlated significantly with clinical symptoms [41]. Overall, these findings support the notion of an immune imbalance phenotype in MDD. Peripheral inflammatory abnormalities may impair dopaminergic system function, potentially through disruption of the blood–brain barrier and activation of central microglia, thereby contributing to the onset and progression of MDD.

Molecular docking and molecular dynamics (MD) simulations provided additional structural-level support for the bioinformatic predictions. GenX formed stable binding conformations with all four target proteins, primarily maintained by hydrogen bonds and hydrophobic interactions. Notably, the GenX-AKR1B1 and GenX-F5 complexes exhibited the lowest binding free energies, suggesting relatively strong binding affinities. The 100 ns MD simulations indicated that all complexes remained structurally stable under physiological conditions, with minimal RMSD fluctuations and balanced system energy. These computational results indicate that GenX could potentially interact with the hub proteins, offering preliminary structural evidence in support of the bioinformatics findings. Nonetheless, the binding and its functional consequences require further experimental validation. Based on the functional characteristics of the four hub genes and the multi-dimensional analyses, we propose a hypothetical pathological model in which GenX may influence energy metabolism, cellular stress, and neuroimmune balance. This model provides a testable framework for future studies. GenX exposure may initially perturb energy metabolism and enhance oxidative stress via downregulation of UCP2 and AKR1B1. Reduced TP53 activity could impair DNA repair and stress responses, while F5 upregulation may contribute to hypercoagulation and peripheral inflammatory activation, potentially compromising the blood–brain barrier and exacerbating neuroinflammation. These interconnected processes might collectively affect dopaminergic neuron energy supply, antioxidant defenses, and synaptic function, potentially leading to reduced dopamine signaling and increased susceptibility to MDD.

However, this study has several limitations: (1) Although the GSE52790 and GSE76826 datasets are representative in terms of serum source, well-defined MDD diagnosis, and comparable whole-transcriptome data, the sample sizes are limited, key clinical covariates (e.g., age, sex, medication use, comorbidities) are lacking, and external validation is not feasible, which may affect the robustness of the findings. (2) A relatively lenient differential expression threshold (|log2FC| > 0.25) was applied to capture subtle transcriptomic changes in peripheral blood, which may increase the risk of false positives. Future studies should incorporate larger sample sizes and multi-omics validation to enhance reliability. (3) The identification of core genes and functional analyses primarily relied on bioinformatics predictions. Although multiple algorithms were used for cross-validation, their functional roles under GenX exposure still require experimental confirmation. For example, knockdown or overexpression of hub genes in cell or animal models could be used to assess effects on neuronal function, inflammatory factor release, and depression-like behaviors, and to validate potential causal relationships among UCP2, AKR1B1, TP53, and F5. (4) Dopamine-related gene selection depended on the GeneCards database. Despite using median correlation scores to improve specificity, over-inclusion is possible, and classical dopamine signaling pathways were not significantly enriched in overall transcriptome analysis. Therefore, interpretations regarding dopaminergic mechanisms should be considered hypothetical rather than directly supported by evidence, and future validation using brain tissue data, cell-type-specific expression profiles, or functional experiments is needed. (5) This study lacks population-based epidemiological evidence linking GenX exposure to MDD risk and cannot directly establish causal relationships among peripheral hub gene expression, GenX exposure levels, and MDD onset. Future research should establish relevant exposure cohorts for further validation. (6) Although machine learning identified TP53, F5, AKR1B1, and other feature genes, the current literature does not support their specific expression or direct functional role in dopaminergic neurons. Therefore, any potential association with dopamine pathways should be regarded as a speculative hypothesis rather than a conclusion directly supported by existing data. (7) Molecular docking and molecular dynamics simulations provide only preliminary structural predictions and lack experimental validation. (8) The ROC analysis mainly assessed the discriminative ability of the hub genes but did not include more comprehensive diagnostic metrics, such as calibration curves, sensitivity/specificity analyses, or confidence intervals. Therefore, the diagnostic performance analysis remains incomplete, and future studies should incorporate these statistical tools to better evaluate model robustness and clinical applicability.

## 5. Conclusions

**Figure 8:**
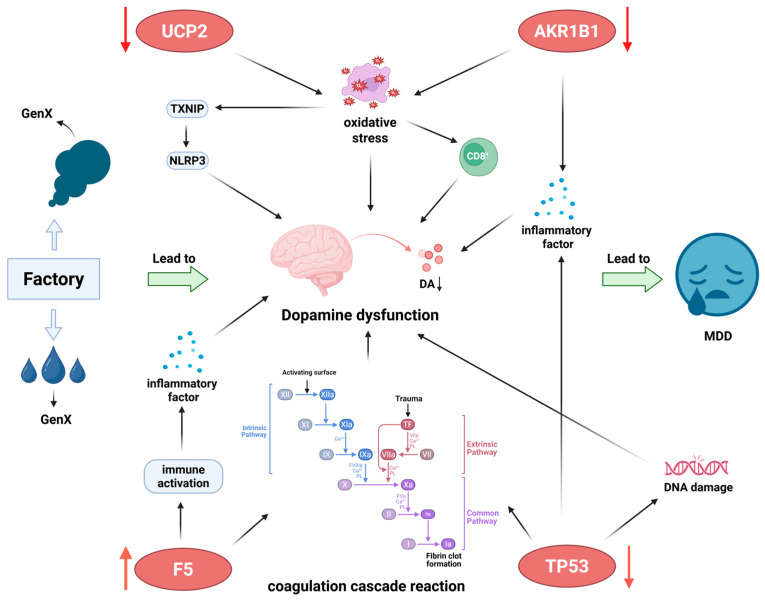
Predicted mechanistic network linking GenX exposure to MDD pathogenesis via the UCP2-AKR1B1-TP53-F5 axis, Created in BioRender 4.2.

In summary, this study integrated multi-omics data and computational biology analyses to construct a potential association map between the environmental pollutant GenX and MDD, and identified core gene modules, including UCP2, AKR1B1, TP53, and F5, that may be involved in this association. Molecular docking and dynamics simulations provided structural-level clues for possible interactions between the proteins encoded by these genes and GenX. Based on the current multi-dimensional analyses, these genes may participate in biological processes related to dopaminergic regulation, such as neuroinflammation, immune-coagulation, cellular stress, and energy metabolism; however, these hypotheses remain speculative and require further experimental validation. Overall, the findings offer preliminary insights into potential links between environmental factors and mental disorder risk and provide directional guidance for future mechanistic studies or preventive strategies targeting these candidate genes (Figure 8).

## Figures and Tables

**Table 1:** Overview of input features, sample distribution, and balancing settings used in LASSO and SVM-RFE.

Module	Input Genes (n)	Balancing Method	Selected Features
LASSO	12	None	8
SVM-RFE (MinError)	12	None	6
SVM-RFE (One-SE)	12	None	6
SVM-RFE (Balanced)	12	None	10

**Table 2:** The list of 12 intersecting genes among GenX-exposure, dopamine-related, and MDD-related gene sets.

Gene Symbol	logFC (in MDD)	Adjusted *p*-Value	Functional Notes
JAK2	0.294	9.40 × 10^−3^	Non-receptor tyrosine kinase; crucial in cytokine signaling and inflammatory responses, which are implicated in MDD pathophysiology.
PHGDH	−0.332	4.89 × 10^−2^	Key enzyme in serine biosynthesis; links metabolic reprogramming to neuronal function and survival.
NT5E (CD73)	−0.747	9.19 × 10^−3^	Ecto-5′-nucleotidase; regulates purinergic signaling, extracellular adenosine levels, and has roles in immune suppression and neuroinflammation.
UCP2	−0.343	4.55 × 10^−3^	Mitochondrial uncoupling protein; regulates energy metabolism, reactive oxygen species (ROS) production, and is neuroprotective.
MFHAS1	−0.273	1.62 × 10^−2^	Involved in innate immune response and regulation of TLR4 signaling; potential link to neuroinflammation.
HSPB1	−0.275	2.45 × 10^−2^	Heat shock protein; functions as a molecular chaperone, protects against oxidative and proteotoxic stress.
AKR1B1	−0.278	4.62 × 10^−3^	Aldo-keto reductase; involved in glucose metabolism, oxidative stress response, and synthesis of inflammatory mediators.
TP53	−0.336	8.17 × 10^−4^	Tumor protein p53; a master regulator of cell cycle, DNA repair, and apoptosis in response to cellular stress.
F5	0.478	1.88 × 10^−2^	Coagulation Factor V; a central player in the coagulation cascade, linking hemostasis to inflammatory processes.
DPP4	−0.445	1.37 × 10^−2^	Dipeptidyl peptidase−4; cleaves neuroactive peptides including GLP−1, and functions as a T-cell activation antigen.
LCK	−0.295	1.11 × 10^−2^	Lymphocyte-specific protein tyrosine kinase; essential for T-cell receptor signaling and adaptive immune activation.
ETS1	−0.344	1.35 × 10^−2^	Transcription factor; regulates differentiation and function of immune cells, including T and B lymphocytes.
JAK2	0.294	9.40 × 10^−3^	Non-receptor tyrosine kinase; crucial in cytokine signaling and inflammatory responses, which are implicated in MDD pathophysiology.
PHGDH	−0.332	4.89 × 10^−2^	Key enzyme in serine biosynthesis; links metabolic reprogramming to neuronal function and survival.
NT5E (CD73)	−0.747	9.19 × 10^−3^	Ecto-5′-nucleotidase; regulates purinergic signaling, extracellular adenosine levels, and has roles in immune suppression and neuroinflammation.
MFHAS1	−0.273	1.62 × 10^−2^	Involved in innate immune response and regulation of TLR4 signaling; potential link to neuroinflammation.

**Table 3:** Molecular docking results of GenX with hub proteins.

Targets of GenX	Alphafold ID/PDB ID	Binding Energy (kcal/mol)
AKR1B1	1US0	−8.2
F5	8TN9	−7.3
TP53	2X0U	−5.8
UCP2	P55851	−6.4

## Data Availability

The original contributions presented in this study are included in the article. Further inquiries can be directed to the corresponding authors.
